# Inhibition of Neointima Hyperplasia, Inflammation, and Reactive Oxygen Species in Balloon-Injured Arteries by HVJ Envelope Vector-Mediated Delivery of Superoxide Dismutase Gene

**DOI:** 10.1007/s12975-018-0660-9

**Published:** 2018-09-06

**Authors:** Shoa-Lin Lin, Jwu-Lai Yeh, Pei-Chia Tsai, Tsung-Hsien Chang, Wei-Chun Huang, Song-Tay Lee, Michael Wassler, Yong-Jian Geng, Erna Sulistyowati

**Affiliations:** 1 Intensive Care Unit, Yuan’s General Hospital, 162, Cheng-Kung First Road, Lingya District, Kaohsiung, 80249 Taiwan; 2 School of Medicine, National Yang-Ming University, Taipei, Taiwan; 3 Department of Pharmacology and Graduate Institute of Medicine, College of Medicine, Kaohsiung Medical University, Kaohsiung, Taiwan; 4 Department of Medical Research, Kaohsiung Medical University Hospital, Kaohsiung, Taiwan; 5 Department of Marine Biotechnology and Resources, National Sun Yat-sen University, Kaohsiung, Taiwan; 6 Intensive Care Unit, Kaohsiung Veterans General Hospital, Kaohsiung, Taiwan; 7 Department of Medical Education and Research, Kaohsiung Veterans General Hospital, Kaohsiung, Taiwan; 8 Department of Medical Laboratory Science and Biotechnology, Chung Hwa University of Medical Technology, Tainan, Taiwan; 9 Department of Biotechnology, Southern Taiwan University of Science and Technology, Tainan, Taiwan; 10 Department of Cardiovascular Biology and Atherosclerosis, University of Texas at Houston, Houston, TX USA; 11 Faculty of Medicine, Islamic University of Malang, Malang, East Java Indonesia

**Keywords:** Gene therapy, Reactive oxygen species, Restenosis, Superoxide dismutase

## Abstract

Extracellular superoxide dismutase (EC-SOD) has been implicated in regulation of vascular function but its underlying molecular mechanism is largely unknown. These two-step experiments investigate whether hemagglutinating virus of Japan envelope (HVJ-E) vector-mediated EC-SOD gene delivery might protect against neointima formation, vascular inflammation, and reactive oxygen species (ROS) generation, and also explore cell growth signaling pathways. The first in-vitro experiment was performed to assess the transfection efficacy and safety of HVJ-E compared to lipofectamine®. Results revealed that HVJ-E has higher transfection efficiency and lower cytotoxicity than those of lipofectamine®. Another in-vivo study initially used balloon denudation to rat carotid artery, then delivered EC-SOD cDNA through the vector of HVJ-E. Arterial section with H&E staining from the animals 14 days after balloon injury showed a significant reduction of intima-to-media area ratio in EC-SOD transfected arteries when compared with control (empty vector-transfected arteries) (*p* < 0.05). Arterial tissue with EC-SOD gene delivery also exhibited lower levels of ROS, as assessed by fluorescent microphotography with dihydroethidium staining. Quantitative RT-PCR revealed that EC-SOD gene delivery significantly diminished mRNA expression of tumor necrosis factor (TNF)-α and interleukin (IL)-1β (*p* < 0.05 in all comparisons). An immunoblotting assay from vascular smooth muscle cell (VSMC) cultures showed that the EC-SOD transfected group attenuated the activation of MEK1/2, ERK1/2, and Akt signaling significantly. In conclusion, EC-SOD overexpression by HVJ-E vector inhibits neointima hyperplasia, inflammation, and ROS level triggered by balloon injury. The modulation of cell growth-signaling pathways by EC-SOD in VSMCs might play an important role in these inhibitory effects.

## Introduction

Increasing evidence shows that inflammation and overformation of superoxide (O^2−^) are important pathological components of neointima hyperplasia [1, 2]. Excessive levels of superoxide may react with nitric oxide (NO) to form reactive nitrogen species that may eventually cause depletion of endogenous vascular NO [3]. Hence, reduction of nitric oxide generation and direct deleterious effects of superoxide may compromise vessel patency [3, 4]. Combating oxidative stress by antioxidant enzymes is important to counteract this process.

Superoxide dismutases (SODs) are a group of antioxidant enzymes that catalyze the dismutation of superoxide radicals into hydrogen peroxide and oxygen. SOD could protect cells from tissue damage associated with the inflammatory process and neutrophil-generated superoxide [5, 6]. Three SODs have been identified, including a copper/zinc containing SOD (CuZn-SOD) which is primarily cytosolic in location, a mitochondrial manganese SOD (Mn-SOD), and extracellular SOD (EC-SOD). EC-SOD is mainly expressed in blood vessels, approximately 50% of the total SOD activity in human aorta is EC-SOD-derived [7]. EC-SOD is mainly synthesized by vascular smooth muscle cells and it is found in inflammatory cells of injured tissue and atherosclerosis [8, 9]. EC-SOD is a secreted enzyme with a long half-life (20 h) in the circulation [10, 11]. It has been implicated in the regulation of vascular function [12, 13]. Several articles have reported the beneficial impact of EC-SOD on vascular remodeling after injury. However, none of these EC-SOD gene delivery experiments [14–17] have explored the cell growth signaling pathway.

Inflammation after vascular injury has been considered an important contributor to atherosclerosis [18].Tumor necrosis factor (TNF)-α, a proinflammatory cytokine, has been reported to be a modulator of leukocyte adhesion and migration in vascular inflammatory diseases. Tumor necrosis factor-α (TNF-α) also has an impact on the regulation of the vascular cell adhesion molecule-1 gene expression in endothelial cells [19]. Another critical proinflammatory cytokine, interleukin (IL)-1β, is one of the known target genes of NF-κB [20]. Previous EC-SOD gene delivery studies have not assessed the effect of EC-SOD in regulation of TNF-α and interleukin-1-beta (IL-1β) expression [14–16, 21]. The impact of EC-SOD gene transfection in regulation of TNF-α and IL-1β expression is worthy of further study.

Hemagglutinating virus of Japan (HVJ) envelope (HVJ-E) is a purified product prepared through complete inactivation of Sendai virus. The HVJ-E vector kit (named GenomOne) has been claimed to be very useful and safe for transfection of molecules into cells and animal tissue by means of membrane fusion. [22] Thus, two-step experiments were performed to investigate (1) the transfection efficacy and safety issue of HVJ-E and (2) to evaluate the impacts of EC-SOD gene delivery in inhibiting neointima hyperplasia, reactive oxygen species (ROS), and vascular inflammation especially on TNF-α and IL-1β expression, as well as the fundamental cell growth signaling pathways.

All forms of reconstruction, whether directly attack the occluding lesion (endarterectomy, angioplasty) or bypass it (vein or prosthetic bypass), cause injury and a wound healing response [23–25]. This wound healing response may lead to luminal narrowing and ultimate failure of the reconstruction [25]. Luminal narrowing or restenosis was the result of excessive intimal hyperplasia, which was a particular problem after carotid endarterectomy [26, 27] or carotid angioplasty and stenting [28]. The aim of this study was to test the hypothesis that gene therapy using EC-SOD gene may have the potential in inhibiting the neointima hyperplasia, which might decrease restenosis after carotid artery reconstruction in patients with severe carotid artery stenosis.

## Materials and Methods

### Animals

The balloon denudation technique to rat carotid artery was similar to our previous method [29, 30]. For anesthesia, 10-week-old male Sprague-Dawley (SD) rats, weighing 350–400 g, were anesthetized with intraperitoneal pentobarbital (50 mg/kg). For euthanasia, the rats were sacrificed by 100% carbon dioxide (CO_2_) inhalation in airtight chambers. The experiments were conducted in accordance with the ARRIVE (Animal Research: Reporting of In Vivo Experiments) guidelines and approved by the Institutional Animal Care and Use Committee (IACUC) of Kaohsiung Veterans General Hospital.

### Materials

The transfection reagent, HVJ-E, was purchased from Cosmo Bio Co., Ltd. (Tokyo, Japan), lipofectamine® was from Thermo Fisher Scientific (Waltham, MA, USA). The human EC-SOD cDNA cloned in pcDNA3.1TOPO expression vector was obtained from the Cardiovascular Biology and Atherosclerosis laboratory of The University of Health Science Center at Houston (Houston, TX, USA). The platelet-derived growth factor-BB (PDGF-BB), and β-actin, dihydroethidum, dimethyl sulfoxide (DMSO) were from Sigma-Aldrich (St. Louis, MO, USA). EC-SOD was obtained from the Cardiovascular Biology and Atherosclerosis laboratory of The University of Health Science Center at Houston (Houston, TX, USA). Anti-rat TNF-α and anti-rat IL-1β were purchased from Novus Biologicals (Littleton, Colorado, USA) and Santa Cruz Biotechnology (Dallas, Texas, USA), respectively.

### Cell Culture

Primary rat vascular smooth muscle cells (VSMCs) were isolated from the thoracic aortas of adult male SD rats similar to our previously described method [29]. Briefly, the isolated thoracic aorta was promptly placed in a cold PBS buffer-filled Petri dish. The fatty tissues, endothelial layer, and adventitial layers of the thoracic aortas were removed using sterile forceps and scissors. Then, the thoracic aorta was cut into small pieces of 1 × 2 mm in size. These tissues were replaced in a tissue culture dish with DMEM containing 10% heat-inactivated fetal bovine serum (FBS), 100 U/mL of penicillin, and 100 g/mL streptomycin, and were maintained at 37 °C in a humidified 5% CO_2_ incubator. When the culture cells reached 70–80% confluence, they were detached using 0.05% trypsin-EDTA for sub-culturing. Cells were cultured in DMEM with 10%. When the culture cells reached 70–80% confluence, they were detached using 0.05% trypsin-EDTA for sub-culturing. Culture media was changed every 3 days and the passage numbers from three to five generations were used for experiments. After synchronization by serum deprivation for 48 h, quiescent VSMCs were stimulated with 10% FBS or 20 ng/mL PDGF-BB for 24 and 48 h, respectively. The quiescent cells cycle were progressively effective for 24 and 48 h stimulation media incubation. HeLa cell line (BCRC 60005, Hsinchu, Taiwan) was cultured in Dulbecco’s modified Eagle’s medium (DMEM) supplemented with 10% fetal calf serum and 1% penicillin streptomycin solution (Gibco, Life Technologies, Carlsbad, CA).

### Preparation and Transfection Using HVJ-E for the In Vitro Study

For the in vitro study, VSMCs of 60% confluence were cultured in serum-free Opti-MEM overnight prior to HVJ-E transfection. Immediately prior to transfection, Opti-MEM was replaced with DMEM containing 10% FBS). The transfection reagent was prepared according to the manufacturer’s protocol. Briefly, the lyophilized HVJ-E was suspended with ice-cooled buffer. The HVJ-E solution (40 μL for each 6-well plate wells) was centrifuged at 13,000 rpm for 5 min at 4 °C. Supernatant was discarded and the pellet was resuspended in 10 μg plasmid, with or without EC-SOD (vector) solution of 1 μg/μL concentration. The plasmid without EC-SOD was regarded as a vector for control study.

### Transfection Efficiency of HVJ-E Measurement

To evaluate the efficiency of HVJ-E vector gene delivery in VSMCs and HeLa cells, the plasmid-encoded green fluorescence protein (GFP) was used as the transfection reporter. In addition, transfections with or without conventional transfection reagent lipofectamine® were used as experimental control groups. The plasmids of pEGFP vector encoding the GFP and pGL3-luciferase (LUC) reporter vector encoding luciferase protein were used to measure transfection efficacy of HVJ-E. Ten micrograms of pEGFP and pGL3-LUC plasmids were transfected into VSMCs and HeLa cells by HVJ-E vector or lipofectamine® reagent, respectively. Lipofectamine® transfection was conducted according to the manufacturer’s instructions. After 24-h transfection, the cells with GFP expression were detected by fluorescence microscopy (AXIO, Zeiss, Oberkochen, Germany) and FACS analysis (BD FACSCalibur, Fitchburg, WI). LUC activity was detected by luciferase assay system (Promega) using a luminescence microplate reader (Berthold Technologies, Bad Wildbad, Germany).

### Cell Viability Assay

According to the manuals from the ATCC and Roche kits, we chose MTT assay for measurement of VSMC and HeLa cells’ viability. VSMCs’ viability was measured by cell counting and by use of the MTT assay kits 24 h after transfection and incubation. Cell viability data are represented as the relative ratio to the serum-free non-transfected group.

### Preparation and Transfection Using HVJ-E for the In Vivo Study

EC-SOD cDNA was inserted into the mammalian expression vector pcDNA3.1D/V5-His-TOPO, and the construct was delivered using the HVJ-E vector. Non-transfected and pcDNA 3.1-TOPO vector-transfected specimens were used as experimental controls. The transfection procedure was performed following manufacturer’s protocol. Briefly, lyophilized HVJ-E powder provided by the manufacturer was suspended with ice-cooled buffer. The 200 μL of HVJ-E solution used for each rat was centrifuged at 13,000 rpm for 5 min at 4 °C. Supernatant was discarded and the pellet was resuspended in 50 μg of either vector or EC-SOD plasmid solutions. Reagent B was added into the DNA-HVJ-E mixture and centrifuged at 13,000 rpm for 5 min at 4 °C to enhance the adhesion of the DNA to the HVJ-E membrane. Supernatant was discarded and the pellet resuspended in buffer combined with Reagent C to increase affinity between the EC-SOD bearing HVJ-E and the cells. The final mixture of 125 μL was injected into the carotid artery and maintained there for 15 min.

### Balloon Injury Model

This study was conducted in accordance with the guidelines of “Kaohsiung Veterans General Hospital Animal Care and Use Committee” under the approved animal study protocol (VGHKS-101-A008). The balloon denudation technique was performed to rat left carotid artery; the right side carotid artery without balloon injury was used as the control. The detail process of surgical method was similar to the method used in our previously reports [29, 30]. Briefly, male Sprague-Dawley (SD) rats weighing 350–400 g were anesthetized with intraperitoneal pentobarbital (50 mg/kg, Sigma-Aldrich, Inc. Missouri, USA), then the left carotid artery was exposed. A Fogarty 2F embolectomy balloon catheter was inserted into the left external carotid artery via arteriotomy and advanced to the aortic arch. The balloon was inflated and withdrawn three times with rotation at the same pressure. The injured segment was clamped with two hemostatic clips on both ends and washed three times with normal saline to remove all residual blood. Plasmid with EC-SOD (*n* = 12) or without EC-SOD (*n* = 12) bearing HVJ-E mixture of 125 μL was injected into the carotid artery and maintained there for 15 min. In other words, the SOD-treated group was the rat treated with EC-SOD gene transfer through HVJ-E (or balloon injury + EC-SOD group). The control groups included (1) the right carotid artery without balloon injury and (2) the left carotid artery received balloon denudation technique and transfected with pcDNA 3.1-TOPO vector (balloon injury + HVJ E vector group) but without SOD gene. Fourteen days after balloon injury, the rats were sacrificed using 100% CO_2_ inhalation in airtight chambers, and sections from both the right and left carotid arteries were excised. The lesion length of balloon injury was about 2.0~2.5 cm. Before preparation, the carotid artery specimen, the portion of each carotid artery with length of 5 mm near the aortic arch, was removed. The reason to discard this most proximal portion was being avoiding the measurement variation due to balloon injury-induced local reaction. The remaining specimen was separated into two parts: the portion close to the aortic arch was prepared for quantitative real-time reverse transcription-polymerase chain reaction (qRT-PCR) and ROS detection studies; the distal portion was fixed with 6% formalin for subsequent analysis. The paraffin-embedded samples were sectioned into 6-μm thickness and used for hematoxylin and eosin (H&E) stain, dihydroethidium (DHE) stain, and immunohistochemistry analysis. The extent of neointimal formation was quantified by computed planimetry of histologically stained sections. The intima-to-media (I/M) area ratio was measured using ImageJ software (NIH, version 1.45).

### Detection of Reactive Oxygen Species

Reactive oxygen species (ROS), such as superoxide, have been proposed to be important signaling molecules in the pathogenesis of intimal thickening in atherosclerosis. To evaluate the effect of HVJ-E-EC EC-SOD in detecting the in situ ROS radical in balloon-injured carotid arteries, the sections were stained with DHE. Carotid artery tissue section from the experimental SD rat was placed on a glass slide. The DHE (10 μM) was topically applied to each tissue section, and covered with a cover slip. Slides were incubated in a dark chamber at 37 °C for 30 min. The tissue section was washed three times in PBS. Fluorescence was detected with a fluorescent microscope (Olympus BX51, Tokyo, Japan) under an excitation wavelength of 535 nm. Fluorescence intensity was measured using ImagingJ software. The extent of fluorescence intensity of the DHE stained cross sections of carotid arteries was expressed as “mean gray levels.” The DHE fluorescence stained area was also measured. It was displayed as the ratio of the cross-sectional area of aorta tissue (including intima, media, and adventitia) to that of outside border of entire aorta (%). The intensities of the DHE fluorescence of the intima and media layers of carotid arteries were also measured.

### Immunohistochemistry

The effect of HVJ-E-EC-SOD on balloon injury-mediated inflammatory response was evaluated by inflammatory cytokines TNF-α and IL-1β detection with immunohistochemistry (IHC) analysis in carotid artery sections. Formalin-fixed, paraffin-embedded core biopsies were sectioned into 6-μm sections and mounted on slides. Following deparaffinization in xylene, slides were dehydrated in an alcohol graded series and placed in running water. The Novolink Polymer Detection System (Leica) was used for immunohistochemistry. The antigen was retrieved by heating in 10 mM citrate buffer (pH 6.0). Slides were then incubated with peroxidase block to neutralize endogenous peroxidase activity, followed by anti-TNF-α (Abcam, Cambidge, MA, USA) and anti-IL-1β (Abcam, Cambidge, MA, USA) antibody (1:100, H00011065-M01, Abnova). Slides were activated with Novolink polymer followed by diaminobenzidine hydrochloride (DAB) chromogen solution to develop peroxidase activity to facilitate visualization of the antibody–DAB complex. Slides were then counterstained with hematoxylin, and the intensities of TNF-α and anti-IL-1β staining measured by ImageJ software (version 1.45). The method for quantifying the intensity of the immunohistochemistry (IHC) staining was similar to the commonly used method of the ImageJ software [31]. The image intensity was defined as “gray level.” The mean gray level was the ratio of the integrated gray levels of total pixels in the region of interest (ROI) divided by the total pixel numbers in same ROI area. Firstly, the ROI of specific area for evaluation was selected by using the ROI Manager. The background image intensity of the cavity of carotid artery was initially determined by measuring the mean gray levels of the cavity area, or “Intensity-1.” Afterwards, we determined the mean gray level of the selected ROI of the outside border of the entire carotid artery tissue of the IHC images, which included IHC staining particles in the carotid artery tissue and the carotid artery cavity (without IHC staining). The resultant mean gray level of the entire ROI was the “Intensity-2.” Thus, the Intensity-2 minus the Intensity-1 was the actual intensity of the IHC staining. The quantifying of the TNF-alpha and IL-1beta staining intensities for individual intima and media layers were also studied.

### Quantitative RT-PCR

Total RNA of the rat carotid arteries was extracted with Trizol reagent (Invitrogen, USA), and the first-strand cDNA synthesized at 42 °C for 60 min using SuperScript VILO cDNA Synthesis Kit (Invitrogen, Carlsbad, CA, USA). The mRNA expression of IL-1β or TNF-α in the rat carotid arteries was evaluated by quantitative real-time reverse transcription-polymerase chain reaction (qRT-PCR) with SYBR Green PCR master Mix (Applied Biosystems, Carlsbad, CA, USA). The sequences of IL-1β primers: 5′-TCT TTGAGGCTGACAGAC-3′ and 5′-CTTGGGTCCTCATCCTGGAA-3′; TNF-α primers: 5′-CCAGGCGGTGTCTGTGCCTC-3′ and 5′-CGACGTGGGCTACGGGCTTG-3′. The temperature conditions of 40 thermal cycles were denaturated at 95 °C for 1 s, with annealing and extension at 60 °C for 20 s. The relative expression levels of TNF-α and IL-1β in rat carotid arteries were calculated by internal control GAPDH. The sequences of GAPDH primers were 5′-GACATGCCGCCTGGAGAAAC-3′ and 5′-AGCCCAGGATGCCCTTTAGT-3′, respectively.

### Western Blot Analysis

After synchronization by serum deprivation for 48 h, quiescent VSMCs were incubated in the absence or presence of EC-SOD gene for 48 h and subsequently divided into three groups: VSMCs without HVJ-E transfection (non-transfected group), VSMCs with HVJ-E but without EC-SOD gene transfection (vector transfected group), and VSMCs with EC-SOD gene transfer through HVJ-E (EC-SOD transfected group). Afterwards, VSMCs were stimulated with 10% FBS (10 min), or 20 ng/mL PDGF-BB (20 min). Reactions were terminated by washing twice with PBS and then homogenized with lysis buffer containing 50 mM Tris-HCl (pH 7.4), 150 mM NaCl, 0.1% Triton X-100, 10% glycerol, 1 mM DTT, 2.5 mM sodium fluoride, 50 μM sodium orthovanadate, 0.5 mM sodium pyrophosphate, 0.5 mM β-glycerophosphate, 1 mM AEBSF, 0.8 μM aprotinin, 50 μM bestatin, 15 μM E-64, 20 μM leupeptin, and 10 μM pepstatin A. Protein concentration was determined using the Bio-Rad protein assay kit and bovine serum albumin (BSA) as a standard. Twenty micrograms of protein were separated on a 10% sodium dodecyl sulphate-polyacrylamide electrophoresis (SDS-PAGE) gel and transferred onto a poly vinylidene fluoride (PVDF) membrane. Western blot analysis was conducted following the manufacturer’s instructions and using specific antibodies against p-Akt, total Akt, p-ERK, total ERK, p-MEK, total MEK (Cell Signaling Technology, Beverly, MA, USA), EC-SOD (Abcam, Cambidge, MA, USA), and β-actin, respectively. Protein was detected with horseradish peroxidase conjugated secondary antibody (Chemicon, USA). At the end of incubation, the membranes were extensively washed with TBS. The immunoreactive bands were detected by chemiluminescence (ECL) reagents and developed by Hyperfilm (GE Healthcare, USA).

### Statistical Analysis

Continuous variable data were expressed as mean ± SD. The statistical significance of the inter-group differences was determined initially by one-way ANOVA with Bonferroni correction for multiple comparisons, followed by unpaired Student’s *t* test. A *p* value < 0.05 was considered statistically significant.

## Results

### Comparison of Transfection Efficacy Between HVJ-E and Lipofectamine®

**Fig. 1:**
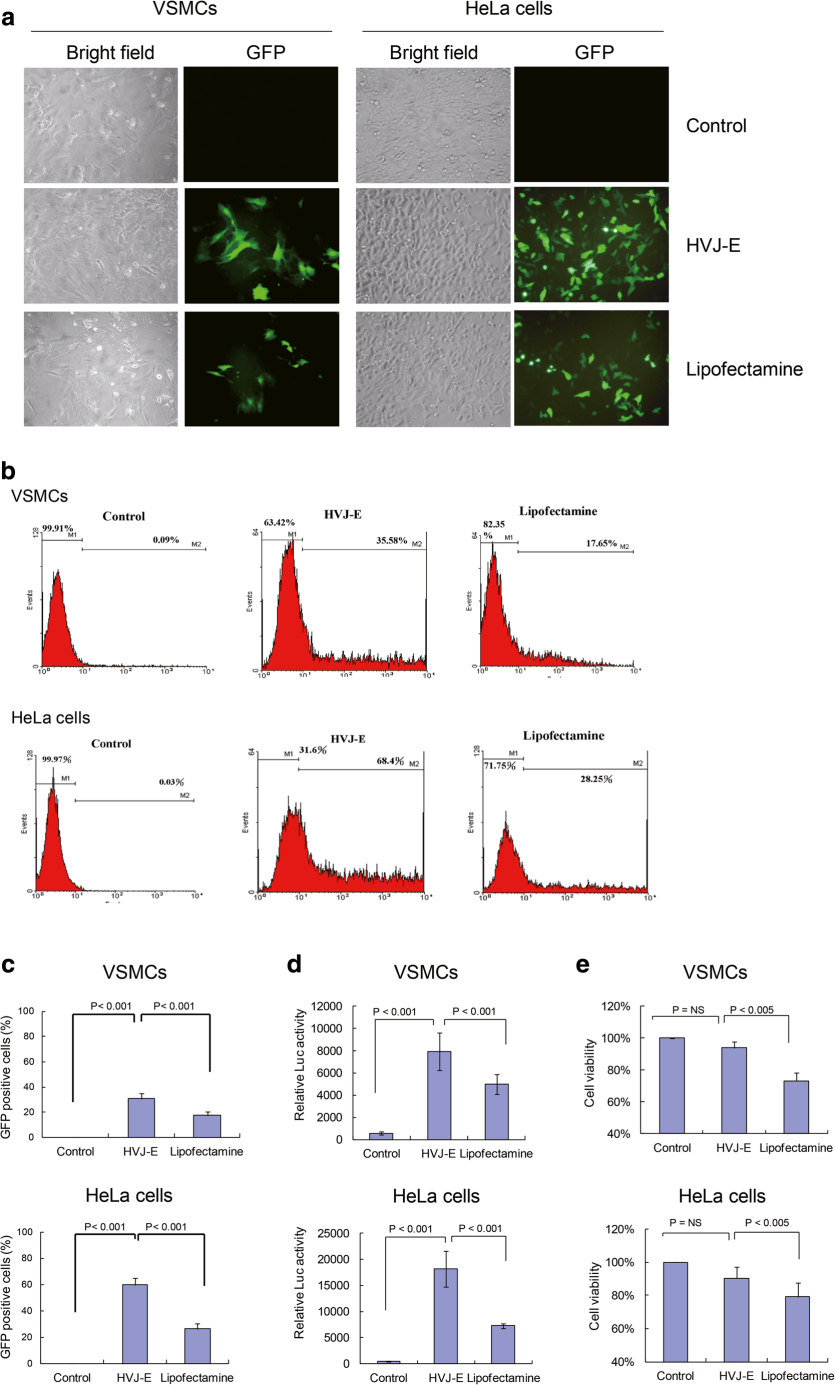
Comparison of transfection efficacy and safety between HVJ-E and lipofectamine. **a** The plasmid encoding green fluorescence protein (GFP) was transfected into VSMCs and HeLa cells with HVJ-E vector, lipofectamine reagent, or without transfection reagent (control). After 24-h transfection, the GFP expression cells (green fluorescence) were captured by a fluorescence microscopy (× 200). The images of bright field were also shown for the comparisons of cell morphology (× 200). **b** FACS data represented the VSMCs (upper panels) and HeLa cells (lower panels) with GFP expression. The percentage of the GFP positive cells was marked as M2. **c** Statistical analysis of FACS data of GFP expression, which depicts the comparison of HVJ-E and lipofectamine transfection efficacy in VSMC and HeLa cells. **d** pGL3-Lucifrease plasmid was transfected into VSMCs and HeLa cells with HVJ-E vector, lipofectamine reagent, or without transfection reagent (control). After 24-h transfection, the luciferase activity was determined using luciferase assay system. **e** The effect of HVJ-E and lipofectamine reagent on VSMCs and HeLa cell viability was evaluated with MTT assay. Data in (**c**–**e)** were obtained from the average of three independent experiments. *VSMCs* vascular smooth muscle cells, *HVJ*-*E* hemagglutinating virus of Japan envelope, *LUC* luciferase

After 24-h transfection, the VSMCs and HeLa cells with GFP expression were observed in HVJ-E and lipofectamine-mediated transfection groups under fluorescence microscopy. From the fluorescence microscopic images, we found that the numbers of green fluorescent cells were much more in the HVJ-E group than that of the lipofectamine® group in both VSMCs and HeLa cells. However, there were no fluorescent cells in the control groups which without transfection reagent. The bright field images showed VSMCs and HeLa cells in three different groups for a comparison under light microscope (Fig. 1a). HVJ-E and lipofectamine®-mediated GFP transfection efficacy were also determined by fluorescence-activated cell sorting (FACS) analysis, which revealed that HVJ-E had significantly higher transfection rates than those of lipofectamine®: approximately 2- and 2.4-fold in VSMCs and HeLa cells, respectively (*p* < 0.001 in both comparisons) (Fig. 1b, c). We also transfected luciferase reporter to compare transfection efficiency between HVJ-E and lipofectamine® in VSMCs and HeLa cells with the reporter assay, which displayed similar results (Fig. 1d). These data suggest that HVJ-E has a higher transfection efficiency than that of lipofectamine®.

### Cell Viability

To determine the safety issue of HVJ-E vector in the gene transfection, the MTT assay was conducted on VSMCs and HeLa cells with HVJ-E and lipofectamine® treatment. HVJ-E treatment revealed significantly greater cell counts compared to those of lipofectamine® treatment (*p* < 0.005 between HVJ-E and lipofectamine treatment in VSMCs; and *p* < 0.001 between HVJ-E and lipofectamine® treatment in HeLa cells, respectively) (Fig. 1e). This result suggests that HVJ-E has a lower cytotoxicity than lipofectamine®.

### HVJ-E-EC-SOD Gene Delivery Inhibits Neointima Hyperplasia

**Fig. 2:**
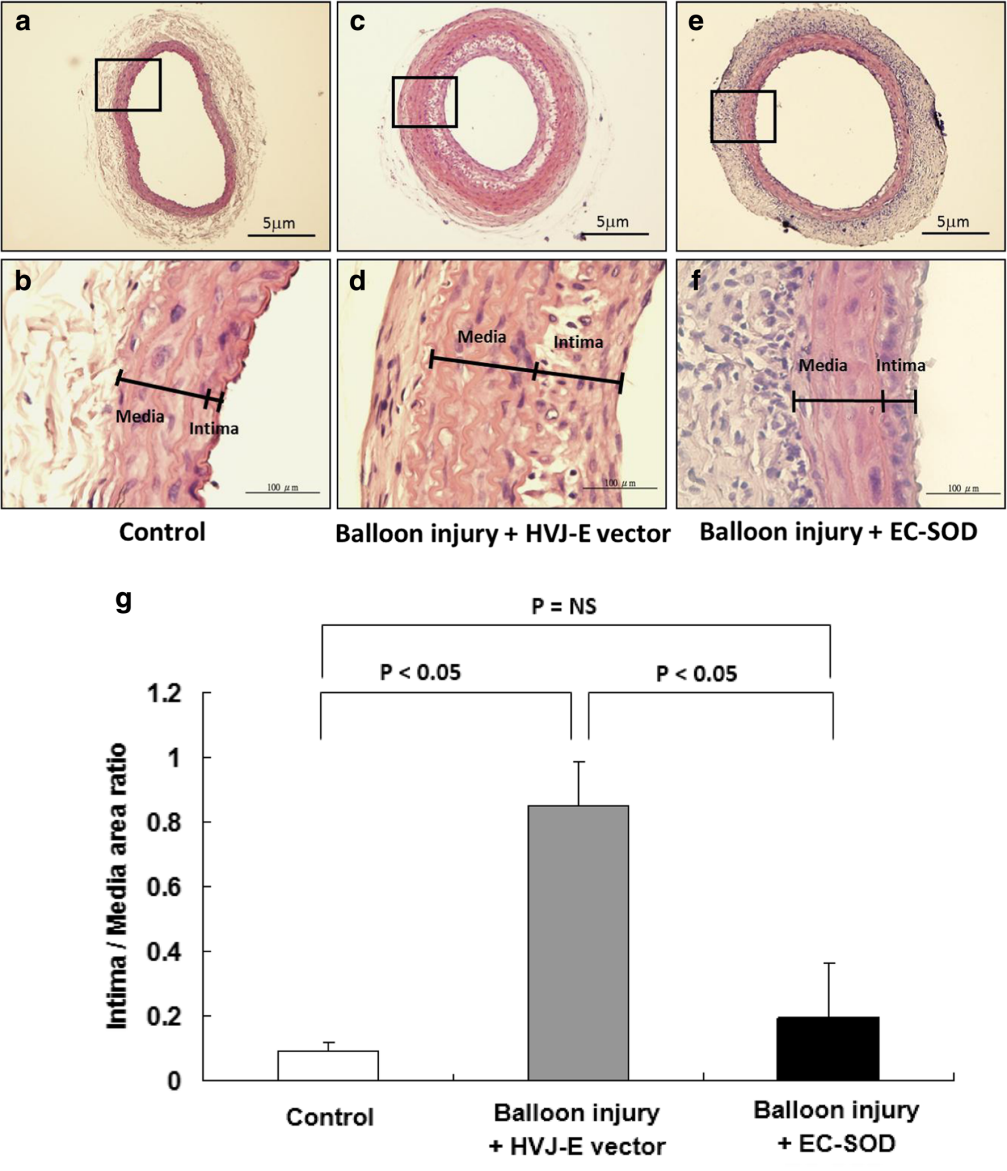
HVJ-E-delivered EC-SOD overexpression inhibited neointima hyperplasia in carotid arteries (**a**–**f**)**.** Hematoxylin and eosin staining images of the carotid arteries of the control group and that of the transfection with HVJ-E-empty vector or HVJ-E-EC-SOD for 14 days after balloon injury are shown. Magnifications for the images was × 40 (**a**, **c**, **e**) or × 400 (**b**, **d**, **f**), respectively. **g** Lower panel was the bar chart demonstrating the effect of EC-SOD on the neointima formation. The area of the intima/media ratio was compared in these three different groups (*n* = 12 for each group). *NS* no significance

Figure 2 shows an example of the cross-sections and morphological analysis of the carotid arteries from SD rats in the control group (Fig. 2a, b) and from rats receiving HVJ-E-vector (Fig. 2c, d) or HVJ-E-EC-SOD transfection (Fig. 2e, f) 14 days after balloon injury. Compared with the vector plasmid-treated (balloon injury) group (Fig. 2c, d), H&E staining showed that balloon injury-induced neointima hyperplasia was attenuated in the HVJ-E-EC-SOD-treated group (Fig. 2e, f). These results revealed that the intima-to-media cross-sectional area ratio (I/M ratio) of the control group (0.09 ± 0.03) was significantly less than that of the balloon injury group (0.85 ± 0.13, *p* < 0.05). Furthermore, the I/M ratio of the EC-SOD-treated group (0.20 ± 0.16) was also significantly lower than that of the balloon injury group (*p* < 0.05) (Fig.2g). These data indicated that the EC-SOD gene delivery slowed down the neointima hyperplasia.

### HVJ-E-EC-SOD Gene Delivery Inhibits ROS

**Fig. 3:**
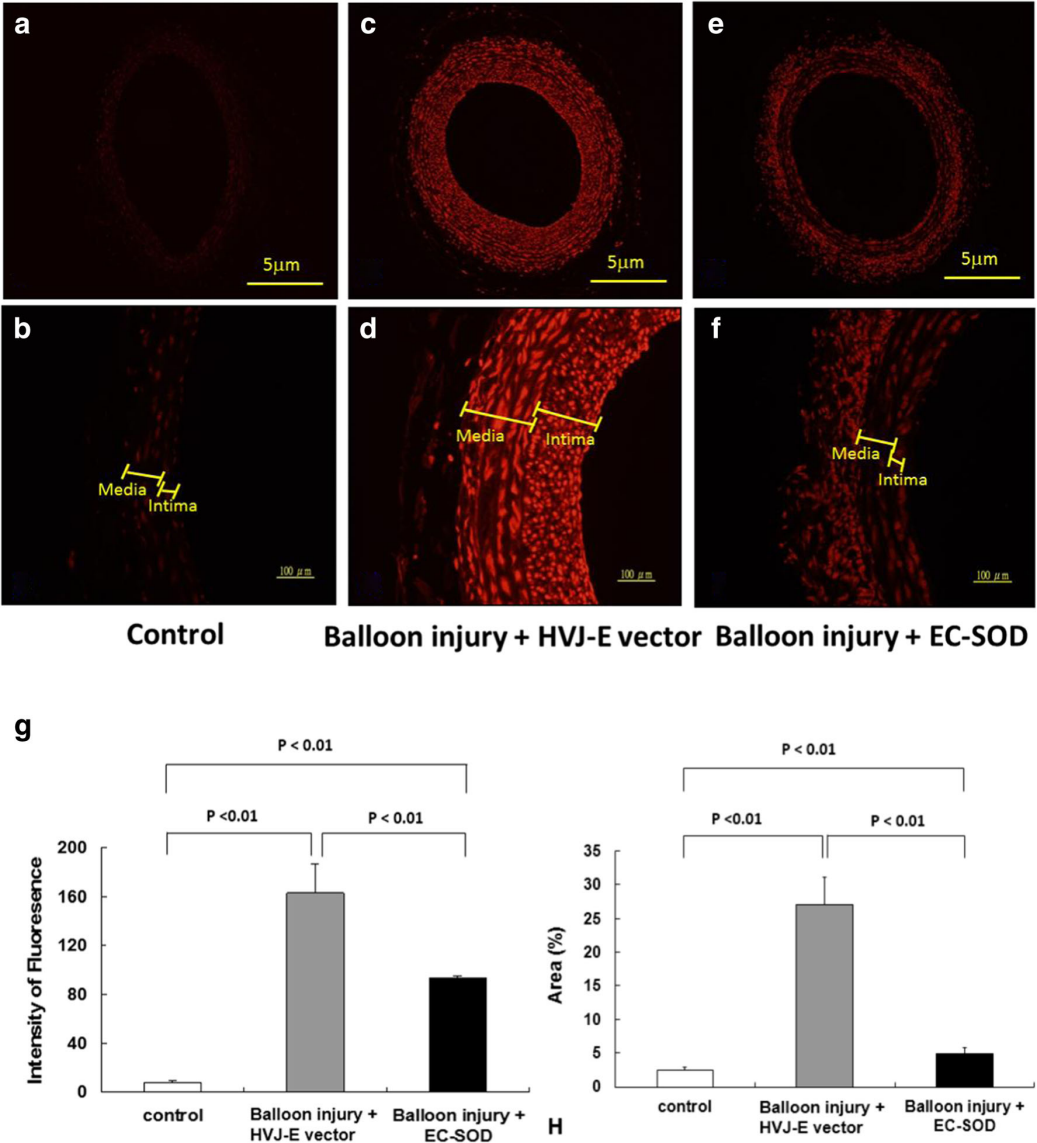
HVJ-E-EC-SOD attenuated the ROS production in balloon*-*injured carotid arteries (**a**–**f**). ROS fluorescence intensity in the carotid artery was evaluated by oxidative fluorescent microphotography using the oxidative fluorescent dye dihydroethidium (DHE). Representative microphotographs showed the carotid artery section without balloon injury (**a**, **b**), 14 days post-balloon injury, with transfection of the HVJ-E-empty vector (**c**, **d**) or with the HVJ-E-delivered EC-SOD (**e**, **f**). **g** Quantitative analysis of the fluorescence intensity (mean gray levels) of the images showed a significant increase in ROS production after balloon injury, compared with the control group or the EC-SOD-transfected group expressed by the DHE staining. **h** Showing similarly findings of the DHE fluorescence-stained area (%) in different groups. (*n* = 12 for each group)

Figure 3 shows an example of microphotography images 14 days after balloon injury with or without HVJ-E-EC-SOD delivery. Compared with the control group (Fig. 3a, b), there was a marked increase in DHE fluorescence, reflecting an increase in ROS radicals in the carotid artery after balloon injury in the “balloon injury + HVJ-E vector group” (Fig. 3c, d). Balloon injury-induced ROS radical level reduced after HVJ-E-EC-SOD transfection (Fig. 3e, f). The EC-SOD-treated group revealed a significant reduction of fluorescence intensity in the carotid artery section when compared with that without EC-SOD transfection (Fig. 3g, *p* < 0.01 in all comparisons) [control group = 7.79 ± 1.57, balloon injury group = 162.91 ± 24.07, (balloon injury + EC-SOD group) = 93.90 ± 1.44, *p* < 0.01 in all comparisons]. The intensities of the DHE fluorescence of the intima and media layers of carotid arteries were 4.9 ± 1.0 vs 4.7 ± 1.2, 158 ± 22 vs 144 ± 28, and 92 ± 5 vs 84 ± 9, respectively of the control, balloon injury + HVJ-E vector, and balloon injury + EC-SOD groups, respectively. (*n* = 6, picture was not shown). The intensities of the DHE fluorescence of the intima layer were greater than those of the media layer, but had not reached statistical significance. Similarly, findings of the DHE fluorescence stained area (%) of different groups were shown in Fig. 3h.

### HVJ-E-EC-SOD Gene Delivery Attenuates TNF-α and IL-1β Protein and mRNA Expression

**Fig. 4:**
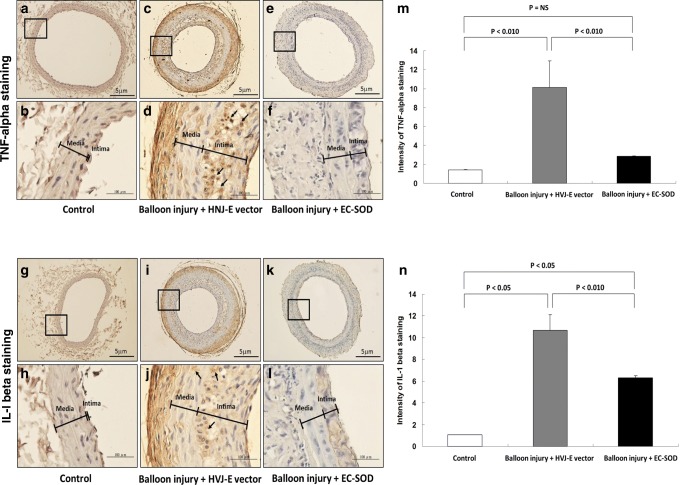
EC-SOD gene therapy decreased TNF-α and IL-1β protein expression in balloon*-*injured carotid arteries (**a**–**l**). IHC images of TNF-α and IL-1β protein expression in carotid artery sections 14 days after balloon injury and EC-SOD transfection are shown. Magnifications for the images was × 40 (TNF-α: **a**, **c**, **e**; IL-1β: **g**, **i**, **k**) or × 400 (TNF-α: **b**, **d**, **f**; IL-1β: **h**, **j**, **l**). TNF-α staining particles (**d**, deep brown, arrows) and IL-1β staining particles (**j**, light brown, arrows) are prominent in the balloon injury group, but much less so in the control group (**b**, **h**) and EC-SOD-treated group (**f**, **l**). Quantitative analysis of the image intensity expressed by the TNF-α and IL-1β staining are shown on **m**, **n**, respectively (*n* = 12 for each group)

IHC images showed that HVJ-E-EC-SOD downregulated the balloon injury-induced TNF-α (Fig. 4a–f) and IL-1β expression (Fig. 4g–l). Quantification of TNF-α and IL-1β IHC signals indicated that the HVJ-E-EC-SOD gene delivery had significant impact on balloon injury-mediated reduction of the carotid artery inflammation (Fig. 4m, n, *p* < 0.01). The intensities of the intima and media layers of carotid arteries were 1.9 ± 0.3 vs 1.6 ± 0.3, 11.0 ± 2.2 vs 9.0 ± 2.6, and 2.4 ± 0.2 vs 2.1 ± 0.2, respectively, for the TNF-alpha staining; and 1.9 ± 0.2 vs 1.7 ± 0.3, 10.5 ± 1.7 vs 9.1 ± 2.0, and 6.8 ± 0.5 vs 6.4 ± 0.7, respectively, for the IL-1beta staining of the control, balloon injury + HVJ-E vector, and balloon injury + EC-SOD groups, respectively (*n* = 6, pictures were not shown). The intensities of the intima layer were greater than those of the media layer, but had not reach statistical significance for both the TNF-alpha and IL-1beta staining images.

**Fig. 5:**
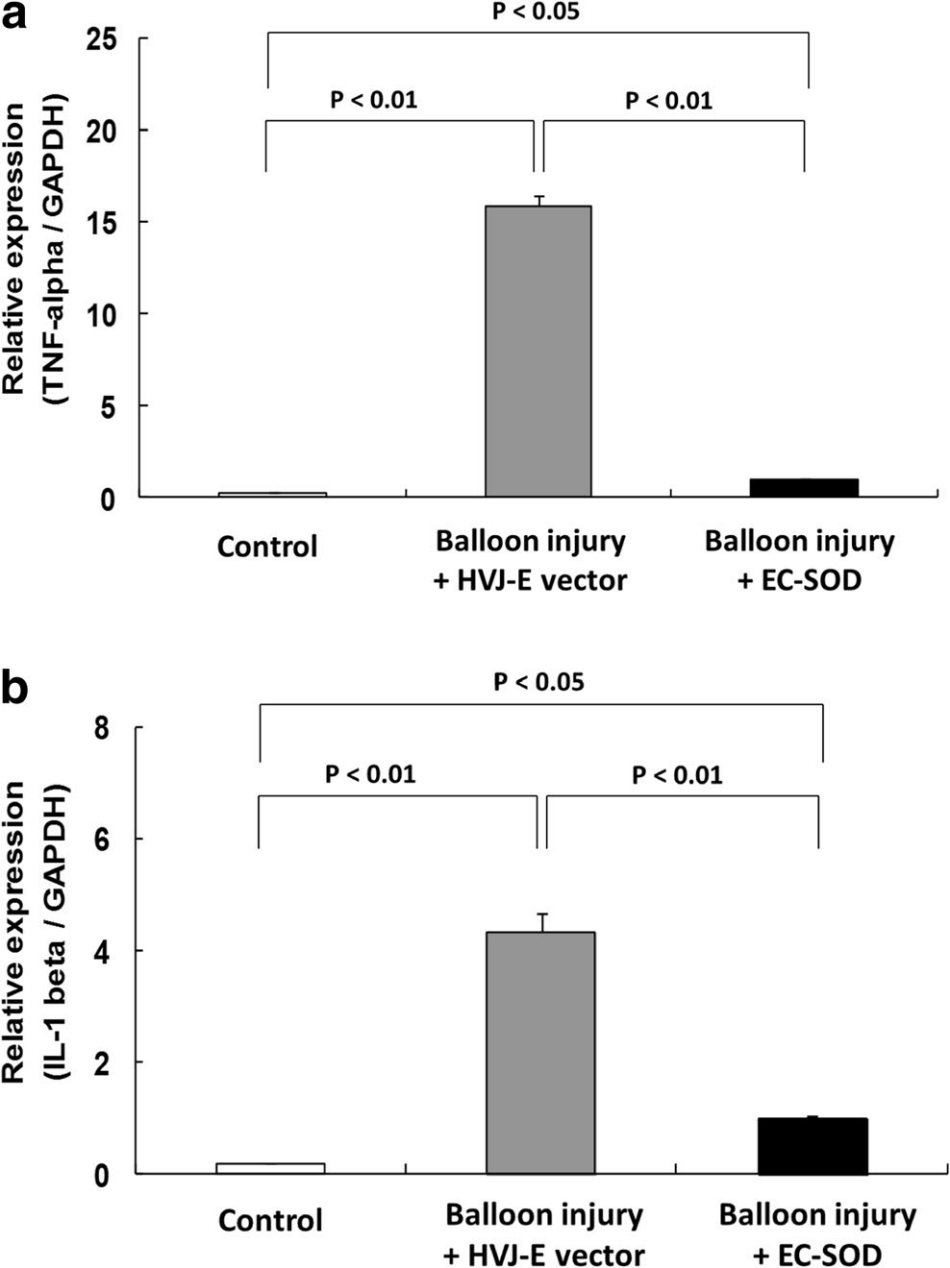
HVJ-E-delivered EC-SOD therapy down-regulated the TNF-α and IL-1β mRNA expression in balloon*-*injured carotid arteries. qRT-PCR analysis revealed that balloon injury significantly increased TNF-α mRNA (upper panel) and IL-1β mRNA expression (lower panel) in local carotid artery tissues, compared with that of control and EC-SOD transfected tissues. Significant inhibition of the inflammatory response after balloon injury was demonstrated after EC-SOD transfection for both TNF-α mRNA (upper panel) and IL-1β mRNA expression (lower panel) (*n* = 12 for each group)

For the qRT-PCR studies, there was a significant increase in TNF-α mRNA expression in the balloon-injured carotid artery tissues (15.92 ± 0.54) compared with that of the control (0.26 ± 0.01, *p* < 0.01) and EC-SOD transfected (1.02 ± 0.02, *p* < 0.01) tissues (Fig. 5a). Similarly, IL-1β mRNA expression was markedly increased in the balloon injury group (4.34 ± 0.34) compared to that of the EC-SOD-transfected group (1.00 ± 0.02, *p* < 0.01) (Fig. 5b). Thus, the results of qRT-PCR investigations suggest that HVJ-E-EC-SOD transfection inhibits inflammatory responses in balloon-injured arteries.

### Effect of EC-SOD on Phosphorylation of ERK, MEK, and Akt

**Fig. 6:**
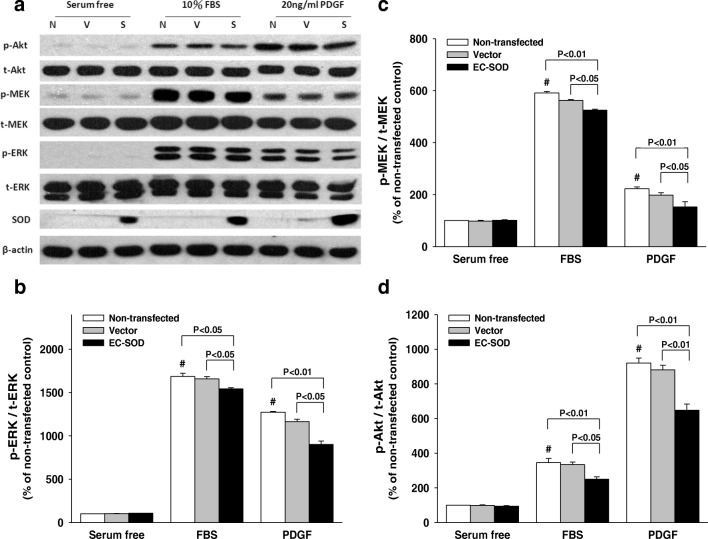
EC-SOD overexpression attenuated FBS or PDGF-induced phosphorylation of ERK1/2, MEK1/2, and Akt in rat VSMCs. **a** Showing the immunoblotting assay. Rat aortic VSMCs were non-transfected (lane N) or transfected by HVJ-E-vector control (lane V) or HVJ-E-EC-SOD (lane S), which were cultured for 48 h, and subsequently stimulated with 10% FBS, 20 ng/mL PDGF-BB, or without stimulation (serum-free condition). Total cell extracts were separated in SDS-PAGE and immunoblotted by antibodies against total (t)- and phosphorylated (p)-Akt, MEK1/2, ERK1/2, EC-SOD, and loading control (β-actin). The quantitative data of p-ERK/t-ERK (**b**), p-MEK/t-MEK (**c**), and p-Akt/t-Akt (**d**) from panel **a** are shown (*n* = 6 for each group)

Western blot analysis of ERK1/2, MEK1/2, and Akt activity and EC-SOD expression can be seen in Fig. 6. Relative protein expression was quantified with a densitometer and is shown in Fig. 6b–d. In Fig. 6a, the data shows that FBS or PDGF-BB induced a profound increase in ERK1/2, MEK1/2, and Akt activation compared to that of the serum-free (SF) condition. Statistical analysis indicates that EC-SOD transfection significantly reduced the level of FBS and PDGF-BB-induced ERK1/2 phosphorylation compared to that of the vector-transfected and non-transfected groups (*p* < 0.05 or *p* < 0.01, respectively) (Fig. 6b). Transfection with EC-SOD significantly decreased the levels of phosphorylated MEK1/2 stimulated by 10% FBS and PDGF-BB (Fig. 6c). Moreover, The EC-SOD-transfected groups had a significantly reduced level of Akt phosphorylation, when compared to that of the non-transfected (*p* < 0.01) and vector transfected (*p* < 0.05) groups after FBS stimulation (Fig. 6d). Similar features could also be found after PDGF-BB stimulation, but the differences were more pronounced (*p* < 0.01 in both comparisons). These data suggest that EC-SOD overexpression may suppress VSMCs proliferation via modulation of extracellular signal-regulated kinase (ERK), MEK, and Akt cell growth-signaling pathways.

## Discussion

Intravenous administration of EC-SOD has been shown to possess no inhibitory effect on the development of neointima hyperplasia [14]. The present study investigated the inhibiting effect of neointima hyperplasia of rat carotid artery after balloon injury by local gene delivery of EC-SOD using HVJ-E vector. We demonstrated that HVJ-E-mediated EC-SOD expression led to a significant reduction of neointima growth, superoxide production, inflammatory response of the TNF-α, and IL-1β mRNA expression in rats with balloon-injured arteries. A mechanistic study in primary VSMCs showed that HVJ-E-EC-SOD downregulated cell growth signaling of ERK1/2, MEK1/2, and Akt activation. Our data suggest that local gene delivery of EC-SOD with HVJ-E vector may be a useful approach to protect against balloon injury-induced artery disease.

Data on the first experiment of this study has verified the transfection efficiency and safety of HVJ-E. It demonstrated that HVJ-E had a better transfection efficacy than that of the conventional vector-lipofectamine® (Fig. 1). The higher cell viability in HVJ-E transfected group might possibly contribute to the increased transfection rate compared to the lipofectamine-transfected group. The fundamental difference between HVJ envelope vector and lipofectamine vector is that HVJ-E vector transfection reagent is a novel and unique transfection material, which employs the membrane fusion ability of the envelope of Sendai virus (Hemagglutinating virus of Japan: HVJ). The genome RNA of HVJ is completely inactivated, so it can be considered to be a “non-viral” transfection reagent with safe and efficiency for transfection use. According to manufactory Manu, HVJ-E vector can be used in vitro and vivo, which was also supported by our results (Fig. 1). Conventional non-viral transfection tools, including lipofectamine or other cationic lipids, are incorporated into cells through endocytosis which results in degradation of most parts of the transferred DNA by lysosomes. On the other hand, HVJ-E vector resists degradation by lysosomes, making it easy to transfer the specified DNA. Therefore, HVJ-E vector yields highly efficient gene expression [31]. Furthermore, sialic acid receptors, which are needed to trigger binding with HN protein, exist in almost all animal cells. Thus, HVJ-E vector is useful for a wide range of targets [32]. After the first in-vitro experiment, we felt more confident about using HVJ-E in the in-vivo study.

There are considerable amounts of literatures targeting neointimal hyperplasia in multiple diverse animal models of balloon angioplasty, which have demonstrated that significant neointimal formation occurred at 10–14 days after balloon angioplasty [33–35]. In these references, neointima was undetectable in the first and third day specimens [35]. Uniform layers of neointima cells first appeared at 7 days and neointima compartment gradually increased thickness from days 7 to 14 [35]. Another animal study found that the relative expression of α-smooth muscle actin in injured segments dramatically decreased from 2 to 4 days after injury and slowly recovered at day 7 reaching the normal level at day 14. As expected, histological examination revealed a significant increase in intimal thickness associated with a slight increase in medial thickness at day 30 post injury. It means that at day 14 post injury, the neointimal formation can be visualized because there are main structural changes in the intimal layer and internal elastic laminae [36]. Since the structural changes of neointimal formation can be well visualized at 14 days post injury and our previous experience found that significant reduction of neointima growth occurred at 2 weeks following carotid artery injury [29, 30]. Thus, this study decided to evaluate the effects of locally administered HVJ-E-SOD 2 weeks after carotid artery injury. We found that HVJ-E-EC-SOD-mediated reduction of neointima growth could be measured at 14 days following carotid artery injury.

ROS are important signaling molecules in the pathogenesis of atherosclerosis and restenosis [37]. ROS production in vascular smooth muscle may contribute to oxidative stress and impaired relaxation in atherosclerotic vessels [38]. Since EC-SOD acts as a superoxide scavenger, which is crucial in maintaining the physical function of cardiovascular system [2], this study provided evidence that EC-SOD gene therapy significantly reduces ROS radicals, as seen in the differences of fluorescence intensities in the arterial tissue sections in comparison with the groups without EC-SOD transfection (Fig. 3). This anti-oxidative effect may be one of the important mechanisms in inhibiting neointima hyperplasia after balloon injury.

Interleukin-1-beta (IL-1β) and tumor necrosis factor alpha (TNF-α) are key cytokines produced by monocytes and activated macrophages. They are the most important mediators of inflammation [39]. This study assesses the effect of EC-SOD overexpression to the balloon injury-induced TNF-α and IL-1β expression using the immunohistochemistry assay. This study found that HVJ-E-EC-SOD gene delivery had significant impact on balloon injury-mediated reduction of the carotid artery inflammation (Fig. 4). This study also assessed the inflammatory response to balloon injury using a quantitative RT-PCR method. We demonstrated that HVJ-E-EC-SOD transfection remarkably inhibits TNF-α and IL-1β mRNA expression (*p* < 0.01) after balloon injury (Fig. 5). These findings indicate that overexpression of EC-SOD by HVJ-E vector has an anti-inflammatory effect on the arterial wall after balloon injury.

This study also evaluated the anti-inflammation effect of EC-SOD transfection after FBS- and PDGF-BB-stimulation using immunoblotting assay. PDGF was released from platelet alpha-granules in response to aggregation and inflammatory stimuli. Secretion of PDGF occurs after adhesion to the injured vessel wall, which is one of the key stimuli for smooth muscle cell migration and subsequent atherosclerosis [40, 41]. The anti-inflammation effect of EC-SOD transfection after PDGF BB-stimulation had not been investigated in previous in-vivo EC-SOD gene transfection experiments [14–16, 21]. The results of this study clearly demonstrate that transfection with EC-SOD is able to reduce FBS- and PDGF BB-stimulated inflammatory response (Fig. 6).

It has been reported that the fetal bovine serum (FBS) is the ideal medium growth supplement and generally preferred over other types of sera. Its high levels of nutrients and optimal combination of growth factors make FBS the most effective growth promoter for virtually any cell culture system [42]. While PDGF-BB, a tyrosine kinase receptor agonist, may induce the proliferation in human smooth muscle cells [43, 44]. Both FBS and PDGF have been used as medium growth supplements for the in-vitro cell culture study. Since our previous experiments have experience in using the 10% FBS and 20 ng/mL PDGF-BB in stimulating the cell growth [29, 30], this study also used similar concentrations, i.e., 10% FBS and 20 ng/mL PDGF-BB.

To the best of our knowledge, most in-vivo cardiovascular EC-SOD gene therapy studies used adenovirus as the vector for gene transfer [14–16, 21, 45, 46]. Using a viral vector for gene therapy may have several disadvantages such as strong immunogenicity [47], more serious side effects, or even death [48, 49]. Here, we used a novel vector—the HVJ-E—as the transfection vector. HVJ-E has been developed as a non-viral vector, consisting of a HVJ envelope but its viral genome is removed [50]. The HVJ-E vector can efficiently deliver therapeutic molecules such as genes, siRNA, decoy oligonucleotides, proteins, and anti-cancer drugs to various tissues in animals and humans with high degree of efficiency [50]. As the viral genome is eliminated in the HVJ-E vector, replication does not occur and viral genes are not expressed in transfected cells [22, 51]. So far, no significant side effects have been reported. Furthermore, it has been claimed that the HVJ-E vector may be superior to presently available vectors for the treatment of intractable human diseases such as bladder carcinoma or melanoma [50].

The current European Society for Vascular Surgery (ESVS) 2017 Clinical Practice Guidelines of management of atherosclerotic carotid disease was published in January, 2018. This report has recommended that in “average surgical risk” patients with an asymptomatic 60–99% carotid artery stenosis, carotid endarterectomy should be considered in the presence of one or more imaging characteristics that may be associated with an increased risk of late ipsilateral stroke, provided documented perioperative stroke/death rates are < 3% and the patient’s life expectancy exceeds 5 years. The carotid stenting may be an alternative treatment to carotid endarterectomy in such patients [52]. The response to the vessel wall injury subsequently triggers platelet aggregation, inflammatory cell infiltration, and proliferation of smooth muscle cells, resulting in restenosis, characterized by neointima hyperplasia [53]. Our study shows that local delivery of EC-SOD gene may contribute to the inhibition of neointima hyperplasia, which might have the potential in decreasing the restenosis after carotid artery reconstruction.

### Study Limitation

There are several limitations that should be mentioned. Firstly, although this study demonstrated that local gene delivery of EC-SOD using HCJ-E vector was capable of inhibiting the neointima hyperplasia and inflammation, whether this method can be applied to clinical use still needs further investigations. Secondly, this study has observed the effect of local gene delivery of EC-SOD at 14 days after balloon injury, but has not assessed whether the inhibiting effect can sustain for a longer period of time. Further investigation for assessing the data after 14 days may be needed. Thirdly, investigation of the ROS and inflammation as well as the TNF-alpha and IL-1beta activities at much earlier time points (before 14 days) may be helpful in understanding the dynamic changes after balloon injury and EC-SOD treatment. However, since the rats’ aortic tissues were small, we only had limited quantity for immunohistochemistry, qRT-PCR, and ROS assay and therefore did not have adequate tissue to study the ROS and inflammation at early stages. It is the limitation of this study. Further investigations are needed to explore the early changes after balloon injury and EC-SOD treatment. Fourthly, although the in-vitro study indirectly demonstrated that EC-SOD gene delivery could significantly attenuate the activation of MEK1/2, ERK1/2, and Akt signaling. It would be interesting to perform further in-vivo study to demonstrate the direct evidence regarding how EC-SOD affecting these signaling activities. However, since the rats’ aorta tissue was limited, we did not have adequate tissue to study these protein signaling. Further in-vivo study is needed.

## Conclusion

This study demonstrates that EC-SOD overexpression using HVJ-E gene delivery has significant effects in reducing the inflammatory response and ROS, and inhibiting the expression of TNF-α and IL-1β cytokines. All of these effects may contribute to the inhibition of neointima hyperplasia after balloon injury. The modulation of cell growth-signaling pathways by EC-SOD in VSMC might play an important role in these inhibitory effects. Local gene therapy using EC-SOD with HVJ-E vector might have potential clinical applications for the prevention of restenosis after carotid artery reconstruction.

## Abbreviations

DHE: Dihydroethidium
ERK: Extracellular signal-regulated kinase
FBS: Fetal bovine serum
HVJ-E: Hemagglutinating virus of Japan envelope
IHC: Immunohistochemistry
IL: Interleukin
MEK: Mitogen-activated protein kinase
NO: Nitric oxide
PDGF-BB: Platelet-derived growth factor-BB chain
SOD: Superoxide dismutase
TNF-α: Tumor necrosis factor-α
VSMCs: Vascular smooth muscle cells
